# Biomechanical Evaluation of Ascending Aortic Aneurysms

**DOI:** 10.1155/2014/820385

**Published:** 2014-06-04

**Authors:** Andrea Avanzini, Davide Battini, Lorenzo Bagozzi, Gianluigi Bisleri

**Affiliations:** ^1^Department of Industrial and Mechanical Engineering, University of Brescia, Via Branze 38, 25123 Brescia, Italy; ^2^Division of Cardiac Surgery, University of Brescia, P.le Spedali Civili 1, 25123 Brescia, Italy

## Abstract

The biomechanical properties of ascending aortic aneurysms were investigated only in the last decade in a limited number of studies. Indeed, in recent years, there has been a growing interest in this field in order to identify new predictive parameters of risk of dissection, which may have clinical relevance. The researches performed so far have been conducted according to the methods used in the study of abdominal aortic aneurysms. In most cases, uniaxial or biaxial tensile tests were used, while in a smaller number of studies other methods, such as opening angle, bulge inflation, and inflation-extension tests, were used. However, parameters and protocols of these tests are at present very heterogeneous in the studies reported in the literature, and, therefore, the results are not comparable and are sometimes conflicting. The purpose of this review then thence to provide a comprehensive analysis of the experimental methodology for determination of biomechanical properties in the specific field of aneurysms of the ascending aorta to allow for better comparison and understanding of the results.

## 1. Introduction

Ascending aortic aneurysms (AsAA) are a life-threatening condition because of the increased risk of aortic dissection. In order to stratify such risk, current guidelines consider the diameter of the aneurismal section as a pivotal parameter for surgical treatment; that is, patients with an ascending aorta dilated to 5.5 cm or greater are recommended for replacement [1].

Nevertheless, it has been previously demonstrated that such parameter may not properly predict the risk of dissection since rupture of AsAA has been documented to occur at diameters less than 4.5 cm [2]. In particular, the concomitant presence of connective tissue disorders (e.g., Marfan syndrome) or congenital cardiovascular anomalies (as bicuspid aortic valve) can lead earlier to aortic dissection. Therefore, the size and diameter of an aneurysm per se cannot reliably predict rupture and dissection.

The possibility to more precisely define the biomechanical properties of the aortic wall has been of increased interest during recent years, in order to achieve an improved understanding of the aortic wall properties and the potential risk for rupture.

The investigations about the biomechanical properties specifically about ascending aorta aneurysms have been approached mostly during the past decade, while there is ample scientific literature with respect to abdominal aortic aneurysms. Moreover, such studies about ascending aorta aneurysms are methodologically heterogeneous, with respect to the biomechanical parameters and the mathematical models utilized in the analysis of aortic wall elasticity: therefore, experimental results are still inconclusive and often conflicting so far [3–6].

The purpose of this review is therefore to provide a systematic overview of the different experimental models utilized to date in order to analyze the biomechanical properties of aneurysms occurring in the ascending aorta.

## 2. Tissue Harvesting and Storing

Mechanical tests on AsAA tissue have been typically performed on specimens obtained from ascending aorta tissue of patients undergoing surgical repair. Usually, tissue is harvested as an intact short tubular structure which is stored and refrigerated at 4°C in saline [3–7], physiological Krebs-Ringer saline [8], or Dulbecco phosphate buffered saline solution without calcium and magnesium [9], either immersed or gauze wetted [10, 11]. Fresh tissue is then tested within 24–48 hours, but there are some notable exceptions to this practice. In the work of Matsumoto et al. [12], specimens were stored frozen at −20°C until measurement. The effects of freezing and ambient temperature were evaluated on specimens from pig thoracic aortas and mechanical properties of frozen-stored specimens at −23°C were almost similar to those of fresh specimens at 37°C.

In the work of Pham et al. [13] instead specimens were harvested over a period of nearly two years and stored fresh by cryopreservation at −80°C, a tissue storage technique which has been recently shown to minimize damage to the elastic components of blood vessels not modifying the biomechanical properties of the elastic arteries [14, 15].

## 3. Specimen Preparation and Preliminary Operations

Specimens for uniaxial tests are obtained by cutting strips along circumferential (C) or longitudinal (L) directions, recording the regions from which they were cut (as anterior, posterior, lateral, or outer/inner). Ideally, specimen should have a high aspect ratio (i.e., ratio between gauge length and width), but the size of the tubular section available actually dictates maximum dimensions. The length of the strips is usually in the order of 30–40 mm and the width is in the order of 8–10 mm [3, 5]. In most cases, particularly when the purpose of the test is the determination of the strength, the middle region of the specimen is narrowed to a width in the range of 2–4 mm [3, 16, 17], although some researchers directly tested rectangular strips [5, 7, 13].

Specimens for biaxial tests are obtained as square specimens aligned with longitudinal and circumferential directions having side length between 15 mm and 25 mm [3, 8, 9, 12, 13]. Square specimen shape is certainly the most common for biaxial testing of biological soft tissue, although some tests on cruciform specimen of pig arteries have also been reported [18].

For bulge inflation test, a slightly bigger dimension (45 × 45 mm) for the specimen has been adopted [19, 20].

## 4. Thickness Measurements

The thickness of the specimens is an important parameter to be accurately measured, since it directly affects stress calculation. Specimens are not perfectly regular, and thickness has been measured in many different ways, including the use of calipers or thickness gauges [5, 11, 13, 17] averaging results at different measurement points or with the specimen sandwiched between two glass slides [3, 9]. Due to low stiffness and thickness of the sample, the contact between caliper and surface must be handled with care, and some researchers have adopted noncontact methods using laser beam micrometers [6, 16, 21]. It should be noted that similar tissues from abdominal aorta aneurysms have been measured using PC-based video-extensometer with a full image CCD camera providing digital images of the sample lying over a counterpart of precisely known height [22].

## 5. Environmental Conditions

With respect to the environmental conditions to allow for optimal tissue hydration during the test, some researchers let the specimen float in a saline bath with slightly different compositions (Krebs-Ringer solution with papaverine, phosphate saline buffer, or Ca^2+^-free and glucose-free Tyrode) either at room temperature [3, 8, 9] or at 37°C [6, 13, 16, 17, 23]. Some other researchers included systems for oxygen or carboxygen bubbling.

Other groups simply maintained the specimen continuously wetted by spraying with phosphate-buffered saline [5, 7, 10, 11]. For bulge inflation test, the specimen remains hydrated on the back-side exposed to the pressure of the water, whereas the outer face is exposed to air at room temperature [19, 20].

## 6. Preconditioning to Test

Before starting the actual test in which measurements are registered, it is a common practice to precondition the biological samples by running a few loading cycles to obtain repeatable stress-strain curves, removing stress relaxation effects, and minimizing tissue hysteresis [11, 13, 17], to recover a condition more similar to the* in vivo* state.

Although all researchers reported this approach and despite the similar type of tissues involved, there is not a common standardized procedure for preconditioning, as can be noticed in Table 1.

Similarly, the load or strain rate applied may be different and is often not comparable across the different studies. In general, the strain rate effect on stress-extension curve and area of the hysteresis loop is considered not considerably wide on soft biological tissues [24].

For AsAA, Okamoto et al. [3] reported that for biaxial testing increasing strain rate from 0.55 to 6% stress-strain curves were very similar. On the other hand, Delgadillo et al. [18] reported instead a significant influence of deformation rate on the uniaxial (and biaxial) response of specimen obtained from pig thoracic aortas, with stiffness of arteries decreasing with increasing deformation rate.

Since overall applied strain rates seem lower than those present in physiological conditions [11], test at higher strain rates could possibly provide new insight into the mechanics and rupture of wall tissue.

## 7. Experimental Methods

### 7.1. Uniaxial Tensile Tests

**Figure 1:**
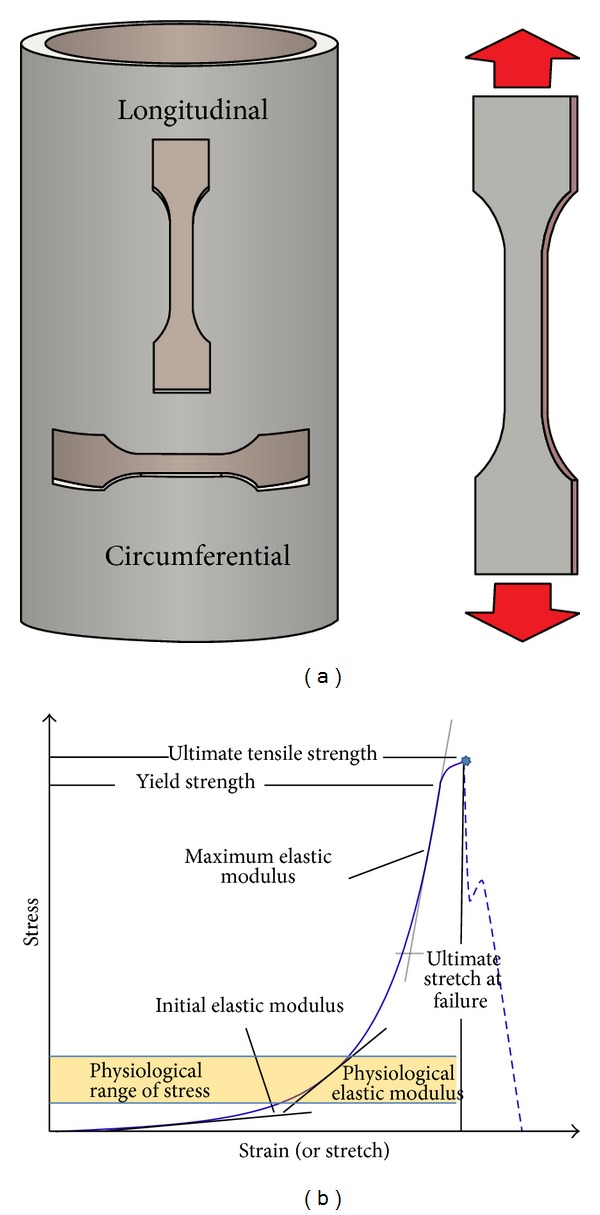
Uniaxial tensile test.

Uniaxial extension is the simplest and most widely utilized method among the* ex vivo* testing methods. A rectangular or dogbone-shaped planar sample (see Figure 1) is subjected to extension along its length at a constant displacement (or load) rate, while the force (or the displacement) is recorded during extension.

When testing biological tissue, special care is needed to limit damage during clamping while avoiding slippage from the grips. The specimen is usually clamped into manual or pneumatic grips using sandpaper and/or cyanoacrylate glues to limit risk of slippage.

Force-extension (F-ΔL) data can then be easily converted into different types of stress and strain measures. Considering works previously mentioned on AsAA, strain is often calculated assuming as initial gage length the distance between the clamps, with the exception of the study of Okamoto et al. [3], in which the displacement of a marker square was tracked with a camera, and of the study of Sokolis et al. [21] in which piezoelectric transducers were glued on the surface of the specimen to measure axial and transverse strains.

A large number of mechanical parameters can be investigated in a tensile uniaxial test, mainly related to failure strength and tissue stiffness. With reference to Figure 1, strength properties are most commonly reported as ultimate tensile strength (*σ*_*f*_) or ultimate stretch at failure (*λ*_*f*_), but a yield strength (*σ*_*y*_) can also be defined as the stress value where the slope of the stress-strain curve starts decreasing.

Concerning stiffness, it is customary, according to classical elastic theories, to express the linear proportionality between stress and strain by defining the Young modulus as the slope of the stress-strain curve in the linear portion of the stress-strain curve.

However, aortic tissue, both healthy and pathological, is largely extensible and exhibits a nonlinear stress-strain response. A single value of elastic modulus does not represent the continuously varying response of the tissue, and the variation of elastic modulus can then be taken into account by introducing an incremental elastic modulus, which is defined by the differentiation of the stress-strain relationship. Values are often provided for specific ranges of stress. Usually, the maximum elastic modulus (MEM), or similar quantities such as peak elastic modulus (*E*_*H*_) or maximum tangential stiffness (MTS), is reported for the failure region. Some researchers also consider a physiological region of the stress-strain curve, identified by the stress range computed using Laplace law for thin tubes for blood pressures between 80 and 130 mmHg [10]. The slope of the curve in this region is then indicated as physiological modulus (PM).

The stretch marking the transition between low and high modulus regions (*λ*_*t*_) is sometimes also reported.

It has to be underlined that there exist many different types of stress (i.e., engineering stress, true or Cauchy stress, and 1st and 2nd Piola-Kirchoff stress) and strain measures (i.e., engineering strain, logarithmic strain, Green-Lagrange strain, and Almansi strain). Each definition may be more or less convenient, depending on which quantities are actually measured during the test, on the range of strain considered (small or finite), and eventually on the constitutive framework in which data will be used (i.e., linear elasticity and hyperelasticity).

Unfortunately, as underlined by Khanafer et al. [25], results presented in the literature for AsAA are rather heterogeneous under this aspect. It would be important for clinicians to have one definition of the stress-strain model to be used to interpret the results of elasticity as this will result in measurements that can be compared.

### 7.2. Biaxial Tensile Tests

**Figure 2:**
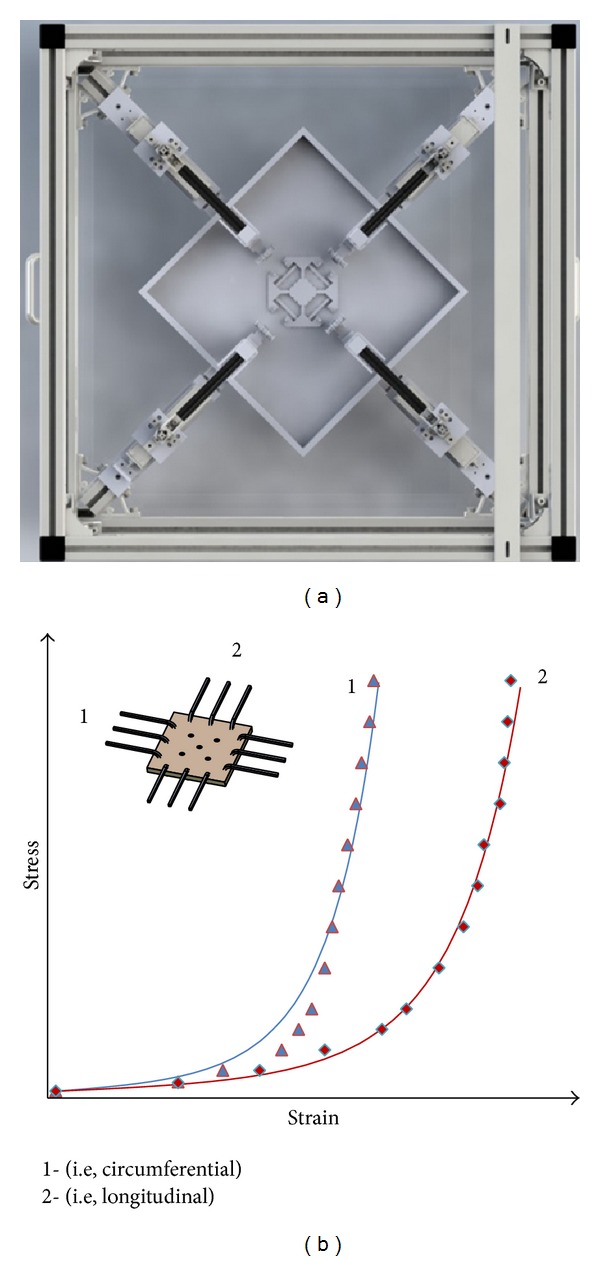
Biaxial tensile test.

Even though uniaxial tensile testing is advantageous for the determination of the failure properties, it may not be the most appropriate methodology for assessing the anisotropy of tissue. In particular, unlike uniaxial testing of circumferential rings or strips, biaxial tests (Figure 2) can be used to determine if tissue properties differ between the circumferential and axial directions. A further advantage is that such investigations are carried out on one single specimen instead of that on two adjacent strips, which could probably have variable composition and biomechanical properties, a consequence of the heterogeneous nature of AsAA wall tissue [21].

On the other hand, aneurysm tissues are stretched biaxially* in vivo*, and there is no guarantee that results obtained from a uniaxial test can be applied to a biaxial state [12]. Since pressure-diameter tests are not well suited to the dilated ascending aorta where the diameter of the specimen may exceed its axial length [3], biaxial experiments are regarded as an alternative way to investigate numerous loading scenarios under physiologically pertinent conditions.

It should be noted however that the “unloaded” reference for stress-strain measurements assumes that the tissue lays flat in its stress-free state. In practice, when testing specimens excised from arteries, some specimens may curl slightly in both the circumferential and axial directions when cut, requiring a small, but unknown, amount of stress to pull the specimen flat [3].

For biaxial tests, most researchers adopted procedures similar to those described by Sacks [26]. The sides of the squared specimen are aligned with the two loading directions. Load is usually applied by means of hooks anchored on each side and connected to the stretcher arms of the test rig by suture wires. The strain is calculated based on video-tracking of the displacement of a square of markers positioned in the central region of the specimen following the procedure originally described by Hoffman and Grigg [27].

By varying applied load ratios, biaxial tests can offer different datasets which could be particularly useful for the purpose of developing advanced constitutive law, allowing for a more reliable computation of material parameter under a multiaxial stress state. As a consequence, biaxial testing has been extensively employed to study the mechanical properties of soft biological tissue and in particular aortic or arterial tissue: healthy, or pathological, or at different locations [28–31].

Conversely, biaxial testing is less suitable for strength assessment, due to attachment techniques and squared shape of the specimen, which may prevent failure of the specimen in the gauge area. Though some have used a cruciform specimen [18] or different types of clamping methods, care should be taken when comparing results obtained under different testing conditions, since geometry and boundary conditions may significantly alter the mechanical response observed in a biaxial test [32, 33].

Results from biaxial tests can be reported graphically in the form of stress-strain curves for the different directions (i.e., circumferential and longitudinal), using Cauchy stress and stretch ratio or Piola-Kirchoff stress measures and Green strains, also depending on the theoretical framework adopted for constitutive modeling. From each individual curve, the same stiffness measures described for uniaxial tests can be obtained. In order to express quantitatively the degree of anisotropy, some authors also introduced an index of anisotropy, but unfortunately there is not a unique definition.

Some used the ratio between different stiffness measurements [8] or normalized moduli in different directions [12], and others considered the ratio between peak Green strains [13] or the ratio between circumferential and axial stress under equibiaxial stretching [3].

### 7.3. Other Test Methods

Uniaxial and biaxial tests have been to date the most common methods used to investigate the mechanical behavior of AsAA tissue, but it is important to underline that other test methods exist and may provide different types of information and new insights into the complex response observed.

#### 7.3.1. Opening Angle Test

Circumferential residual stresses are present in arterial walls, and one of the methods to characterize them is to cut radially a ring-shaped specimen and measure the opening angle formed by the specimen as it own accord opens. For a detailed description of the test method and of the relevant literature for its interpretation, the interested reader can find more information in researches by Labrosse et al. [34, 35].

For the specific case of AsAA, the only research using this method was carried out by Okamoto et al. [3]. Starting from a ring of tissue 3–5 mm wide placed in room-temperature saline, they compared digital images of the closed ring with images taken during the opening process, which takes place as soon as ring is cut radially, until a stable configuration was reached after 5–7 min. Overall, they found that opening angle was significantly higher for older patients, with mean values ranging from about 200° to 260°.

Although the opening process is an indirect evidence of the presence of residual stresses in the intact ring, the interpretation of results and the incorporation into a material constitutive model are not trivial, and some have questioned about the possibility to characterize the residual stress state in an artery by means of a single parameter such as the opening angle [36].

#### 7.3.2. Bulge Inflation Test

Bulge inflation test is another technique that allows for investigation of biaxial mechanical behavior, which has been applied in the past to the study of human and pig aortas [37, 38]. The basic testing protocol consists in obtaining square specimens from an excised cylindrical aortic tissue laid flat. These are successively clamped in the inflation device forming a hermetically sealed cavity in which a fluid (water) is injected at controllable rate simultaneously measuring pressure.

The inflation of the strip is tracked optically, and when rupture occurs, a measure of tissue strength can be obtained.

This method has been recently extended to AsAA tissues [19, 20], in particular to test single media and adventitia layers. Stereo-digital image correlation (SDIC) was used to obtain the strain field of the whole inflated membrane, combined with the application of the virtual field method (VFM) to calculate associated the stress field. Although the implementation of such methods requires considerable expertise, the application of such advanced digital imaging techniques can significantly broaden the potential scope of the test, allowing simultaneous evaluation of material parameters for constitutive modeling purposes and of localized stress in the area that eventually ruptures.

#### 7.3.3. Inflation-Extension Test

Mechanical properties of blood vessels have been studied by pressurization of a whole cylindrical segment. Several works can be found in the literature for both human and pig aortas (see, among others, the study by Labrosse et al. [34, 35] and Kim and Baek [39], for example, and the relevant literatures). Video-based tracking technique with multiple markers embedded or affixed to the specimen is typically applied in order to enable monitoring of the large deformation of the vascular tissue.

Combined extension and inflation of a segment of an artery (a circular cylindrical tube) provide data that are equivalent to data obtained from planar biaxial tests [40]. However, biaxial testing removes the sample curvature and does not take into consideration the residual stress characterized by the opening of an unpressurized aortic segment when it is cut longitudinally. On the other hand, pressurization of a blood vessel segment preserves sample curvature and can include residual stress analysis as well. The inflation-extension test has thus been considered by many as the preferred test for estimating* in vivo* stress, also because it closely reflects the motion of the aortic wall during the cardiac cycle [39].

Yet, nonstandardized, complex protocols, where the sample is successively pressurized at different fixed lengths, or under different axial loads, make this inflation-extension technique potentially difficult [35].

To date, there is no evidence of application of this test to the case of AsAA, possibly also because of the limited size and curvature of the tubular specimen available from surgical repairs. It should be mentioned however that for AAA a new experimental setup has been recently implemented to precisely measure the deformations of an entire pressurized model of abdominal aortic aneurysm by means of a stereoscopic imaging system utilizing two cameras to measure model aneurysm displacement in response to pressurization [41].

#### 7.3.4. Other Methods

Other advanced experimental methods were recently applied to healthy or pathological aorta. These include nanoindentation test to determine multilayer material properties of aorta to explain local mechanisms of deformation, force transmission, tear propagation and failure in arteries [42], the development of novel biaxial tensile test for studying aortic failure phenomena at a microscopic level [43], and the use of 4D ultrasound data for* in vivo* determination of elastic properties of the human aorta [44].

Finally, some research groups are working in the direction of evaluating layer specific properties [45], including analysis of fiber orientation, or dissection properties by means of peel test to assess delamination strength of arteries [22]. Their application to AsAA would certainly be beneficial to provide new insight into mechanical properties.

## 8. Test Protocols

A summary of some of the main characteristics of different test protocols for AsAA adopted by various research groups is presented in Table 1. As it can be noticed, due to the lack of standardized procedures and test protocols, similar types of test have been conducted and reported in different ways, adding a further variable that makes even more difficult the interpretation and comparison of data from different sources.

## 9. Histological Analysis

In most studies reported so far, histological analysis has been performed in order to correlate mechanical properties of aortic wall to pathologic findings. In most cases, authors obtain specimens of wall adjacent to sections used for biomechanical testing, in order to minimize bias associated with anisotropy [4].

Concerning the preparation of tissue analysis with optical microscopy, samples were fixed with formalin solution at 10% for 24 hours, dehydrated with ethanol or other alcoholic solutions at increasing concentrations, and embedded in paraffin wax. Subsequently, they were sectioned in 3 to 5 *μ*m thick histological slides.

The stains utilized in such studies had a dual purpose: the classical stain with hematoxylin-eosin was adopted for an overall tissue analysis, while other specific stains for the individual constituents were used for percentage quantification of the same. In particular, orcein [6], Hart's elastin [4], Verhoeff's Van Gieson [13], Movat pentachrome [8], and Sirius red [6] have been used to chromatically differentiate the relative presence of elastin, collagen, smooth muscle cells, and mucoid material.

For precise percentage quantification of these components, dedicated software has been used, which is able to detect color differences through spectrometric analysis, by integration with relieving optical systems mounted on optical microscope [6, 8].

## 10. Conclusions

From a biomechanical point of view, rupture events occur when the stresses acting within the walls as a result of blood flow and boundary conditions exceed the strength of the wall tissue. Calculating the risk of rupture therefore requires the prediction, by means of analytical or finite element (FE) models, of the tensile stresses on the aneurysm's wall and knowledge of the corresponding failure stress levels. This approach basically replicates the one adopted in the field of engineering structural mechanics, in which failure criteria are employed to rationally compare stress state components with material strength.

However, the unique structure of biological tissues makes its application to the prediction of aneurysm rupture not as straightforward as one may hope. On the one hand, the properties of aneurismal tissue are difficult to determine, may vary significantly on an individual basis as well as regionally (i.e., depending on the location considered on the aneurysm), and may evolve with time in response to changes on the surrounding environment or as consequence of pathological conditions. Thus, assessing the strength of the tissue as a patient specific value is still an open critical issue.

On the other hand, biological tissues exhibit a complex behavior in which the distribution, arrangement, and proportion of elastin and collagen fibers may result in highly nonlinear and anisotropic characteristics. Complex mathematical models of the tissue capable of reproducing its biomechanical properties in the context of FE models are therefore necessary in order to accurately compute the wall tension throughout the entire aneurysm. Such analysis is further complicated by the need of a precise description of the three-dimensional geometry of the aneurysm (including wall thickness) and boundary conditions.

Experimental techniques may help in this sense, since they can potentially provide some answers to both of these critical issues and improve our understanding of the failure process.

The mechanical properties of AsAA tissue have been investigated by several groups in the course of the last ten years, using different approaches which may vary depending on the purpose of the test and samples availability.

Overall, the experimental data available to date for AsAA are still limited, since despite the importance of wall strength in the mechanics underlying the aortic failure, in general, there has been less attention to that of thoracic aortic aneurysm in comparison with abdominal ones [46, 47].

Most of the published data refer to uniaxial or biaxial tensile tests on strips excided from dilated or aneurismal thoracic ascending aorta. The mechanical behavior and strength of dilated human ascending aorta were first investigated by Okamoto et al. [3, 4] with a series of uniaxial, biaxial, and opening angle tests and by Vorp et al. [5] by means of uniaxial tests in longitudinal and circumferential directions.

Biaxial tensile properties were the object of experimental tests in works by Choudhury et al. [8], Matsumoto et al. [12], and Fukui et al. [48], in which local responses of healthy and diseased human ascending aorta were compared. Iliopoulous et al. [6, 16] carried out a campaign of uniaxial tests to investigate stiffening and weakening aspects of AsAA walls and the influence of regional and directional variations. Further investigations concerning elastic modulus at different pressure, again based on uniaxial tensile tests, were instead presented by Duprey et al. [10] and Khanafer et al. [11]. Iliopoulos et al. later reported a mathematical characterization of AsAA wall [23] and the characterization of single layers of AsAA walls by uniaxial test [21]. A comparison of the mechanical properties of healthy and diseased human ascending aortic wall has been also provided by García-Herrera et al. [17].

Considering the most recent works, biaxial (and uniaxial) tests were described by Pham et al. [13], evaluating the influence of bicuspid aortic valve (BAV) and bovine aortic arch (BAA) and using results as a base to develop a new predictive rupture potential [49]. Biaxial test data were also provided by Azadani et al. [9], whereas in the study by Pichamuthu et al. [7] uniaxial response on AsAA walls was investigated as a function on aortic valve phenotype and collagen content. A rather different and innovative experimental approach has been instead described by Romo et al. [19] and Kim et al. [20], involving bulge inflation test on planar specimens, the use of stereo-digital image correlation to investigate the strain field, and the application of the virtual fields methods for material characterization purposes.

Finally, the dependency of rupture properties on gender was recently studied by Sokolis and Iliopoulos [50], showing that,, in uniaxial testing up to failure, male AsAA were stronger but equally extensible in the circumferential axis compared to female ones. Longitudinally, instead, gender differences at each region were insignificant.

All researches reported were based on tissue data from* ex vivo* tests. Isolating samples may also introduce as yet unknown changes to their behavior affecting the results of* in vitro*/*ex vivo* tests. To determine mechanical properties of aneurismal tissues,* in vivo* “tests,” involving the use of ultrasound phase-locked echo-tracking MRI (b) or time resolved ECG-gated CT imaging, have also been adopted for abdominal aortic aneurysms in particular to evaluate the compliance of the aneurismal wall [47]. Although* in vivo* tests do reflect the actual response of the living tissue, from a biomechanical point of view the main difficulties are accurately determining the true force and the displacement distribution and ascertaining stress-free configuration of the biological entity. This limits their usability for constitutive modeling purposes; moreover, these investigations on tissue strength are quite obviously not possible.

As a consequence,* in vitro*/*ex vivo* mechanical testing on samples excised from a tissue is currently the most common source of quantitative data for both tissue strength assessment and stress-strain response in a form useful for the development of advanced constitutive equations.

## Figures and Tables

**Table 1:** Summary of test procedures.

Authors	Environmental condition^3^	Preconditioning		Strain or loading rate	Stress measure^1^	Strain measure^2^
		*N*° cycles	Range			
Uniaxial						
Okamoto et al., 2002 [3]	Bath, RT	10	0.1–0.49 N	0.59 N/s	*σ*	E, *λ*
Vorp et al., 2003 [5]	Wetting, RT	10	0–7% strain	8.5%/min	*σ*	e, *λ*
Iliopoulos et al., 2009 [6]	Bath, 37°C	Y, ∗	—	20%/min	*σ*	E, A
Matsumoto et al., 2009 [12]	Bath, RT	No	—	0.2 mm/s	*σ*	E
Duprey et al., 2010 [10]	Wetting, RT	2	0-1 N	10 mm/min	*σ*	*ε*
Khanafer et al., 2011 [11]	Wetting, RT	10	—	10 mm/min	*σ*	*ε*
Iliopoulos et al., 2011 [23]	Bath, 37°C	Y, ∗	—	20%/min	*σ*, 2^PK^	*λ*, E
Sokolis et al., 2012 [21]	Bath, 37°C	Y, ∗	—	20%/min	2^PK^	E
García-Herrera et al., 2012 [17]	Bath, 37°C	5	30% of max. load	0.03 mm/s (18%/min)	*σ*	*λ*
Pichamuthu et al., 2013 [7]	Wetting, RT	10	0–7% strain	8.5%/min	*σ*	e, *λ*
Biaxial						
Okamoto et al., 2002 [3]	Bath, RT	10	10% equi-stretch	2–4%/s	*σ*	E
Matsumoto et al., 2009 [12]	Bath, RT/37°C	N	—	0.2 mm/s (1.7%/s)	*σ*	E, *λ*
Choudhury et al., 2009 [8]	Bath, RT	10	5 mm disp.	0.1 mm/s	s, ∗	e, ∗
Pham et al., 2013 [13]	Bath, 37°C	40	Equi-stress	∗	1^PK^, 2^PK^	E
Azadani et al., 2013 [9]	Bath	10	10% equi-stretch	Waveform, 0.5 Hz	*σ*	E

^1^s: engineering stress, *σ*: true or Cauchy stress, and 2^PK^: second Piola-Kirchoff stress; ^2^e: engineering strain, *ε*: true or logarithmic strain, E: Green strain, *λ*: stretch ratio, and A: Almansi strain; ^3^RT: room temperature; ∗: details not specified.
